# Exploring what lies behind public preferences for avoiding health losses caused by lapses in healthcare safety and patient lifestyle choices

**DOI:** 10.1186/1472-6963-13-249

**Published:** 2013-07-02

**Authors:** Jeshika Singh, Louise Longworth, Amanda Baine, Joanne Lord, Shepley Orr, Martin Buxton

**Affiliations:** 1 Multidisciplinary Assessment of Technology Centre for Healthcare (MATCH), Department of Information Systems and Computing, Brunel University, Uxbridge, Middlesex UB8 3PH, UK; 2 Health Economics Research Group (HERG), Brunel University, Uxbridge, Middlesex UB8 3PH, UK; 3 Department of Civil, Environmental and Geomatic Engineering, University College London, London WC1E 6BT, UK

**Keywords:** Health care safety, Stated preferences, Hypothetical scenarios, Priority setting

## Abstract

**Background:**

Although many studies have identified public preferences for prioritising health care interventions based on characteristics of recipient or care, very few of them have examined the reasons for the stated preferences. We conducted an on-line person trade-off (PTO) study (N=1030) to investigate whether the public attach a premium to the avoidance of ill health associated with alternative types of responsibilities: lapses in healthcare safety, those caused by individual action or lifestyle choice; or genetic conditions. We found that the public gave higher priority to prevention of harm in a hospital setting such as preventing hospital associated infections than genetic disorder but drug administration errors were valued similar to genetic disorders. Prevention of staff injuries, lifestyle diseases and sports injuries, were given lower priority. In this paper we aim to understand the reasoning behind the responses by analysing comments provided by respondents to the PTO questions.

**Method:**

A majority of the respondents who participated in the survey provided brief comments explaining preferences in free text responses following PTO questions. This qualitative data was transformed into explicit codes conveying similar meanings. An overall coding framework was developed and a reliability test was carried out. Recurrent patterns were identified in each preference group. Comments which challenged the assumptions of hypothetical scenarios were also investigated.

**Results:**

NHS causation of illness and a duty of care were the most cited reasons to prioritise lapses in healthcare safety. Personal responsibility dominated responses for lifestyle related contexts, and many respondents mentioned that health loss was the result of the individual’s choice to engage in risky behaviour. A small proportion of responses questioned the assumptions underlying the PTO questions. However excluding these from the main analysis did not affect the conclusions.

**Conclusion:**

Although some responses indicated misunderstanding or rejection of assumptions we put forward, the results were still robust. The reasons put forward for responses differed between comparisons but responsibility was the most frequently cited. Most preference elicitation studies only focus on eliciting numerical valuations but allowing for qualitative data can augment understanding of preferences as well as verifying results.

## Background

Studies examining public preferences have found that people consider factors in addition to direct health costs and consequences when asked for their opinions regarding resource prioritisation in health care [1-3]. Prevention of harm from lapses in healthcare safety is an area which the public might choose to prioritise even if it leads to no greater health gain than another preventative healthcare service [4]. In contrast people may give less priority to health loss when the individual is considered responsible for their own condition [5,6].

We conducted a study to elicit relative valuations placed on different types of healthcare interventions to prevent lapses in healthcare safety, prevent genetic disorders and discourage illness associated with lifestyle choices [7]. Lapses in healthcare safety were described in terms of responsibility for the ill health and could be perceived as attributed to the NHS system or staff. Where responsibility was perceived to lie with the NHS, we additionally explored prioritising the avoidance of health losses for NHS staff compared to patients. The scenarios included: hospital associated infections, medication errors and NHS staff injury. Other contexts related to lifestyle choices: lifestyle disease and sports injuries. Finally, a context for which responsibility could not be attributed to either the individual or the healthcare system (genetic disorders) was chosen as the basecase for comparison. Values were elicited using a person trade off (PTO) method which adopts an explicitly societal perspective rather than an individual choice perspective [8-10]. The respondents were presented with hypothetical paired choice question between interventions and asked to assume they were equivalent in terms of cost and health gain per patient, and only differed in terms of ‘responsibility’ and the number of patients benefitting.

The focus of the survey was on collecting quantitative data, but we were keen to understand the reasons for stated preferences and at the end of every PTO question, we asked the respondent to comment why each choice was made. Commenting on the choice was optional.

The study found that members of general public attached a premium to spending addressing hospital associated infections when compared to genetic disorders; drug administration errors were valued similarly to genetic disorders; and NHS staff injuries were valued slightly lower. Lifestyle diseases and sports injuries, for both of which the individual has some responsibility, were given the least priority. The results suggested that responsibility does matter to people, but that preferences are complex, as the people differentiate within the same category of responsibility. Multivariate analysis suggested that preferences may be related to individuals’ perceptions of their exposure to the different types of risk and fear of their consequences [7]. However, these factors explained only a small part of the wide variations in stated preferences.

This paper aims to identify the considerations behind respondents’ stated preferences by examining their comments, and presenting the reasons for each comparison. It is unclear whether the differences result from different understandings or interpretations of the questions asked, and this will be tackled in our analyses. Most preference elicitation studies only focus on eliciting numerical valuations but this is an important study which steps out further to understand why preferences were made.

## Methods

### Study design

The survey was conducted in September 2010 using a self-completion questionnaire administered over the internet following approval from the Brunel University Research Ethics Committee. The methods of the survey have been reported in detail elsewhere [7]. In brief, the sample consisted of 1030 members of general public and quotas were employed to obtain a sample that reflected the UK national population by age, gender, geographical location and occupation based on 2001 UK Census.

Six different types of healthcare services were included which resulted in fifteen possible combinations. The first five comparisons used genetic disorder as a baseline. The five comparators were: lifestyle related diseases, sports injuries, hospital acquired infections, medication errors and injuries to NHS staff. The sixth scenario was a random pairing of any of the conditions. For each PTO question, respondents were asked ‘why did you make that choice?’ and had the option of ticking ‘no comment’ or writing an explanation for the choice in the free text space provided. The questionnaire also collected information about respondents’ socio-economic status, their use of and attitudes towards the NHS; and their perception of the level of risk, dread, and experience in each of the six contexts.

The scenarios were illustrated with examples of the type of risk that the services were seeking to avoid, and the type of service that they might include. The risk examples were chosen to avoid particularly emotive risks such as cancer or childhood illnesses, for example diseases caused by smoking or drinking too much (lifestyle related disease) inherited eczema or high blood pressure (genetic disorder). The services were all presented as simple preventive programmes rather than treatment programmes, for example devices with built-in safety features to prevent needles stick injuries (NHS staff); safer sports equipment to prevent back injury (sports injury); computerised prescribing to prevent patients being given wrong drugs (medication errors). All of the services were targeted at protecting NHS patients, apart from category six, which was directed at NHS staff.

The PTO valuation questions were framed as a policy choice, in which respondents were asked to imagine that their local health service had some additional money to spend on preventive health care within their area. They were given two alternatives to choose from and informed that the local health service could only afford one of them: for example a service to prevent lifestyle related disease and a service to prevent diseases due to genetic disorders. All the health services were explicitly stated to avoid identical health losses per person (3 months of moderate ill health) and to cost equal amounts of money to the NHS (£200,000 per service). Respondents were presented with two services, A and B, which would benefit 1,000 people each, and asked to choose between the two, or to indicate indifference. A full example of the PTO questions used is illustrated elsewhere [7].

If participants were indifferent, it was assumed that they valued both services equally. Where the participant indicated a preference for option A or B, they were asked how many more people would need to benefit from the less preferred intervention in order for them to give equal priority to the interventions. The number of people benefitting was varied until indifference was achieved and strength of preference was drawn. The participants also had the option of specifying their own value or of stating that the NHS should never prioritise the less preferred service, indicating a refusal to trade [7]. After every PTO question the respondents were asked why they made their choice.

### Analysis of qualitative data

The comments explaining the respondents’ preferences in the PTO questions formed a large qualitative dataset in terms of number but the responses were brief. A thematic analysis framework was adopted whereby qualitative data was transformed into explicit codes conveying similar meanings [11,12]. A step-by-step process was used for this thematic content analysis and involved three distinct stages [11]. The first stage was familiarisation, where the aim was to obtain an overview of some of the broader themes [13]. In the second stage, coding took place and general themes were named and defined. The coding helped in making the data manageable or amenable to analysis [13]. Microsoft Excel was used to aid coding and later, was used to filter, categorise and analyse the trends in responses.

Finally the themes were aggregated to form an overall coding framework in order to develop the analyses in the end. Two broad categories were identified: 1) factors which determined preferences; and 2) factors which challenged the assumption stating that all contexts would incur equal cost and identical health loss avoided per patient affected as described in scenarios. The study reflected the main ideas put forward based on their frequency or dominance [12].

We also conducted reliability tests to check for bias or inconsistencies in the primary coder’s assessment of the comments. Random samples of 20 comments for each comparison (100 in total) were coded separately by a second reviewer. Differences in coding were discussed between the reviewers, and after agreement of how to apply the coding, all of the comments were re-coded. The process was repeated a second time or until acceptable inter-rater agreement was established. Intercoder agreement and Cohen’s Kappa test were used to estimate inter-rater agreement.

To assist with the interpretation of the data, the qualitative comments and application of the coding framework were considered for categories defined according to their response to the quantitative questions. For example, whether they preferred interventions to prevent NHS system failures, preferred lifestyle interventions, were indifferent, and so on. Most comparisons included the prevention of illness resulting from genetic disorders; however additional questions with a comparator selected from the other conditions were also examined. There was little data on these comparisons and the analysis focussed on interventions for conditions with potentially similar perceptions relating to responsibility: lifestyle conditions and sports injuries; NHS system and staff errors.

## Results

### Quantitative results

The quantitative results have been reported in full elsewhere [7]. However to place the qualitative data in context, we have summarised the PTO responses in Table 1a. Respondents were grouped into five categories based on their preferences;

**Table 1:** Summary of quantitative data

**Summary of PTO data (a)**						
**Context**	**Non-trade genetic**	**Prefer genetic**	**Indifferent**	**Prefer comparator**	**Non-trade comparator**	**Total**
**Lifestyle related diseases**	257 (25%)	368 (36%)	220 (21%)	144 (14%)	41 (4%)	1030
**Sports injuries**	384 (37%)	450 (44%)	149 (14%)	35 (3%)	12 (1%)	1030
**Hospital infections**	68 (7%)	127 (12%)	405 (39%)	268 (26%)	162 (16%)	1030
**Medication errors**	159 (15%)	208 (20%)	313 (30%)	208 (20%)	142 (14%)	1030
**Staff injuries**	280 (27%)	311 (30%)	273 (27%)	89 (9%)	77 (7%)	1030
**Total**	1148	1464	1360	744	434	5150
***Summary of PTO data excluding comments which challenged PTO assumptions in respective context (b)***						
*Lifestyle related diseases*	*251 (26%)*	*353 (37%)*	*218 (23%)*	*109 (11%)*	*32 (3%)*	*963*
*Sports injuries*	*347 (37%)*	*403 (43%)*	*147 (16%)*	*28 (3%)*	*9 (1%)*	*934*
*Hospital infections*	*57 (6%)*	*107 (12%)*	*399 (44%)*	*212 (23%)*	*131 (14%)*	*906*
*Medication errors*	*136 (16%)*	*160 (18%)*	*308 (35%)*	*156 (18%)*	*116 (13%)*	*876*
*Staff injuries*	*255 (27%)*	*267 (28%)*	*273 (29%)*	*85 (9%)*	*73 (8%)*	*953*
	*874*	*999*	*850*	*465*	*308*	*4632*
***Summary of PTO data excluding respondents who challenged PTO assumption once or more (c)***						
*Lifestyle related diseases*	*167 (24%)*	*238 (35%)*	*146 (21%)*	*107 (16%)*	*26 (4%)*	*684*
*Sports injuries*	*253 (37%)*	*291 (43%)*	*107 (16%)*	*22 (3%)*	*11 (2%)*	*684*
*Hospital infections*	*39 (14%)*	*83 (12%)*	*266 (39%)*	*187 (27%)*	*109 (16%)*	*684*
*Medication errors*	*99 (14%)*	*145 (21%)*	*211 (31%)*	*135 (20%)*	*94 (14%)*	*684*
*Staff injuries*	*185 (27%)*	*207 (30%)*	*180 (26%)*	*57 (8%)*	*55 (8%)*	*684*
	*743*	*964*	*910*	*508*	*295*	*3420*

•‘non-trade genetic’: these are the respondents who had dominant preference for genetic disorder and indicated that NHS should always prioritise it over the competing service. In other words, they would never trade one patient in the genetic group for any number of patients in the comparator group.

•‘prefer genetic’: this group preferred prevention of genetic disorder over the comparator and indicated strength of preference in the trade off exercise.

•‘indifferent’: these respondents considered the two interventions to be equal.

•‘prefer comparator’: this group preferred the non basecase context and indicated that the NHS should always prioritise it over the base case.

•‘non-trader comparator’: this group indicated dominant preference for the non basecase context and indicated strength of preference in the trade off exercise.

### Qualitative dataset

Some of the respondents’ comments are quoted verbatim below to give a real flavour of the responses received. They are quoted exactly, inclusive of typographical errors. Out of the 1030 respondents, 90% provided at least one comment. Frequencies of comments provided for each type of question are presented in Table 2. Overall ‘no comment’ was given in only 23% of the 5150 PTO questions. Upon closer examination, another 6% of comments were excluded from the analysis as they did not provide any explanation for the choice that was made, or the comment was incomprehensible. They were grouped together as ‘no reason provided’, for example:

**Table 2:** Summary of qualitative data

**Context**	**No reason provided**	**Miscellaneous**	**Inconsistent**	**Usable comments**	**Number of codes**
**Lifestyle related diseases**	212	39	23	756	832
**Sports injuries**	277	24	2	727	819
**Hospital infections**	314	23	4	689	830
**Medication errors**	328	11	0	691	792
**Staff injuries**	366	13	3	648	774
**Total**	1497	110	32	3511	4047

(ID 1738) hi.

(ID 5951) Because thats the way I believe it should be.

In addition, a few comments were ‘inconsistent’, as they referred to a context not referred to in the question. For example, a respondent would refer to a hospital infection when the question was asking about sports injuries against genetic disorders.

There were also some ‘miscellaneous’ comments which provided a vague explanation for the choice made by the participant, but did not fit into any other code category or required further interpretation by the reviewer which could be subjective.

(ID 1638) Nhs staff can claim compensation if they get an injury at work.But with all the equipment they have these days for lifting patients etc.which are one off items that hospitals can run charity events to raise money .genetic related comes first.

(ID 3228) The hospital know best benefits of treatment to patients.

All of these comments were excluded and the remaining 68% with sufficiently clear explanation were included. A total of 3511 comments were included for analysis. Multiple codes could be attributed to each comment and 4047 different codes were identified from those comments.

### Coding framework

**Figure 1:**
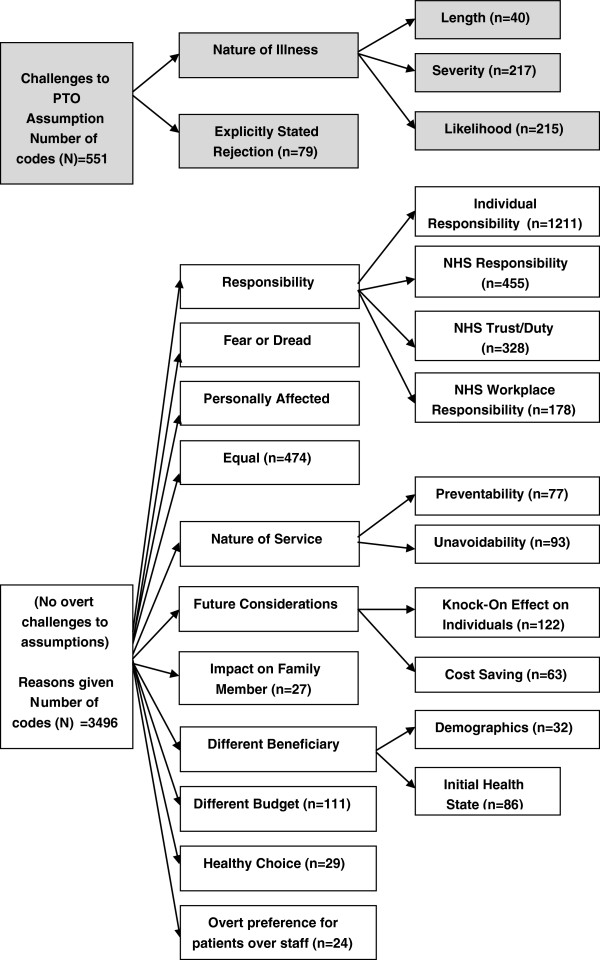
Coding framework.

Once individual codes were established for each context, they were put into an overall framework that aimed to help us understand how the codes related to each other. Many comments suggested that respondents preferred one type of preventative service over another because of misunderstanding the scenario or not accepting the assumption of equal costs and benefits across services. The coding framework was used to separate excerpts which explicitly contradicted the assumptions of the hypothetical choices we put forth regarding identical cost and benefits of all six interventions. It is illustrated in Figure 1.

Overall there were two overarching themes which contradicted the assumptions of hypothetical choice scenarios presented and consisted of 14% of total codes applied. First there were excerpts which challenged the assumption of equal health loss avoided and referred to the probability, duration or severity of the adverse event. Some respondents reported one type of condition to be more severe than the other, despite being told in the hypothetical choice question that all patients would experience 3 months in a moderate health state without the intervention. Differences in the likelihood of illness was also noted although each choice question specified that the number of people who would benefit (or be affected) from the services as identical for all contexts. Secondly there were excerpts which explicitly rejected that the contexts were of equal cost or equal benefit. Only three respondents posted excerpts which challenged the assumptions for all five questions against basecase.

The summary of quantitative data, firstly excluding the responses which challenged the assumption and secondly excluding all respondents who ever challenged the assumptions, is presented in Table 1b and c. The comparison indicates that excluding these data from main analysis does not affect the results drawn, as the distribution of preferences does not vary.

For the first reliability test whereby a second reviewer coded a total of 100 comments, the inter-coder agreement between the two reviewers was 77%. Upon completion of the second reliability test an inter-coder agreement of 87.5% was reached. Cohen’s Kappa test estimated a Kappa agreement of 66.3%, which is termed as ‘substantial agreement’ between reviewers.

### Explanatory comments by preferences

**Figure 2:**
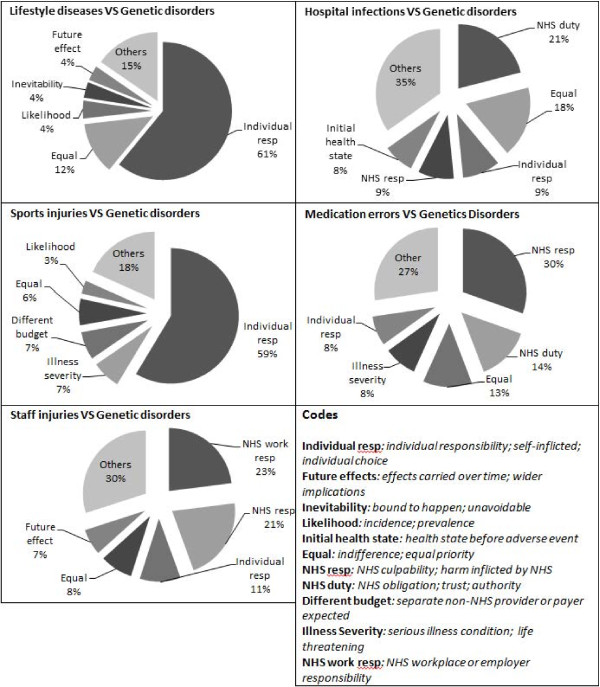
Reasons given for each context.

The prominent themes for each comparison against basecase are reported in Figure 2. Below, we examine the reasons provided for each preference group: indifferent, prefer (prevention of harm from) genetic disorder, prefer lifestyle related disease, prefer sports injuries, prefer hospital associated infection, prefer medication error and prefer NHS staff injuries.

### Indifferent

Almost all respondents who indicated indifference or equal priority to the preventative services being compared commented that the contexts were equal. Some cited they were equal in terms of costs, some mentioned they were equal in terms of benefit and some mentioned both. This suggested that respondents focussed only on the stated health benefits and/or costs, and characteristics of the recipient or intervention did not affect their response.

However some respondents noted similarities in terms of responsibility, apart from costs and consequences. Many mentioned individual and NHS responsibility (or lack of) when comparing preventative services for prioritisation, for example:

(ID 413) Whilst some may argue that lifestyle is a choice and a genetic disordeer is not - I believe it is not as simple as that. Lifestyle ‘choice’ may not be a choice at all.

(ID 293) In both cases the patient has no control, therefore equal attention or service must be provided.

There were also few instances where the respondent was indifferent between interventions because of personal bias such as being at higher risk or having family members experiencing both the diseases.

Finally, a few responses comparing sports injuries with genetic disorder noted that although it is self-inflicted and genetic disorder is not, “(ID 1195) sport is exercise which is good for the body and people should not be penalised for trying to stay fit”; so they chose to value both equally. Similarly in the case of staff injuries, there was an appreciation of their service which made respondents reluctant to choose the comparator, for example “(ID 1144) NHS staff are doing an extremely valuable service and deserve to be protected in the place of work. Genetic diseases are not brought on through a particular lifestyle so should be of equal importance to provide a certain quality of life.”

### Preference for preventing genetic disorders

Genetic disorder was used as the basecase in the study and all remaining services were compared against it. A majority of respondents preferring prevention of genetic disorders cited that genetic disorders were nobody’s fault, and lacked individual responsibility. Most of the respondents who prioritised preventing genetic disorder over lifestyle related diseases stated that they blamed the individual for inflicting lifestyle diseases and said that it was their own choice and expressed sympathy for those with genetic disorder for not having any control over it. Similarly when genetic disorders were compared against staff injuries, many argued that such injuries are a workplace hazard and the staff members would be aware of the risks of the chosen job. Verbatim responses included:

(ID 63) It’s out of our hands if a genetic disease but lifestyle choices are the individual’s responsibility and not the nations.

(ID 1138) Genetically inherited diseases are entirely haphazard in affecting patients whereas lifestyle related diseases are to some extent a known outcome of certain behaviour.

(ID 564) staff should know risks!

A number of responses noted the inevitability of genetic disorders. They argued that prevention of illness related to genetic disorders should be prioritised because they are unavoidable and require a collective and systemic approach. A few respondents who preferred genetic disorders over sports injuries or medication errors added that it was an are requiring further research.

When medication errors and hospital associated costs were compared against genetic disorders, some of the respondents prioritised preventing the latter but their explanations contradicted. They considered safety a fundamental component of NHS services and not an option, for example:

(ID 341) Preventing hospital borne diseases should be an automatic, it shouldn’t get a priority because it should (not) happen at all.

(ID 970) ’service to prevent patients being given incorrect drugs’ should be an essential and ensured part of any medical staff training, and should not be required as a separate process.

(ID 7632) Surely preventing wrong drugs being given should not require extra services. It should be a matter of course of being a nurse/doctor. If that is not the case then the individual concerned should not be employed as they are clearly not up to the job.

Some respondents preferred to prevent genetic disorder over sports injuries, because they felt sports injuries were beyond the NHS remit and a few suggested using private insurance to cover treatment when required. For example:

(ID 4095) Prevention of sports injuries should be the responsibility of the relevant sport’s governing body, not the NHS.

(ID 251) if one participates in sport then they should take out an insurance policy to cover any injuries.

(ID 3985) Sports Injuries should not be funded by the NHS. Where treatment is received in an NHS facility (in cases of emergency) it should be paid for.

Similar views were expressed regarding staff injuries, medication errors and hospital associated infections where they were categorised as a ‘management’ issue and one that should receive separate funding. A few noted that staff injuries were an “in-house” problem and expressed an overt preference for preventing illness in patients over staff.

Finally some respondents preferred to prevent genetic disorders over sports injuries, staff injuries and medication errors because they stated they were more prevalent or would result in more severe consequences. These responses challenged the assumptions of the hypothetical setting we presented which stated identical health effect and number of people affected for all the services.

### Prefer preventing lifestyle-related diseases

Some were keen to prioritise prevention of lifestyle-related illness because they perceived *genetic disorders will happen anyway,* while lifestyle related diseases were easier to prevent.

(ID 600) Genetic disorders cannot be completely prevented, because they are genetic, you are born pre-disposed to that disorder. lifestyle can be altered.

(ID 383) Because if people can be persuaded to change their lifestyle it could mean fewer having to be treated.

Some respondents noted that lifestyle related diseases have externalities, for example a healthier lifestyle will be passed on to children. Similarly it was noted that for genetic disorders future generations will benefit. A few responses mentioned personal experience as the reason of preference:

(ID 2108) because i have a disease due to my lifestyle and if there had been more help when i needed it i might not be in the position that im in today.

A few responses indicated preference for lifestyle-related diseases because they thought they were more prevalent than genetic disorders.

### Preferred preventing sports injuries

Only a small number of respondents preferred prevention of sports injuries over genetic disorders. A few respondents who valued sports injuries more argued that while sports injuries may be the result of an individual choice, at least that person is trying to keep active and stay healthy in the choices that they make.

More responses indicated that they did not accept that the two preventive services for genetic disorder and sports injuries were of equal cost and would avoid identical health loss compared to the other scenarios. These respondents stated that sports injuries were less severe than genetic illnesses and added *sports injuries are not life threatening*. Other respondents mentioned sports injuries are less prevalent than genetic disorders. Some also argued that sports injuries are easier to prevent, than the comparator.

### Preferred preventing hospital associated infections

While some respondents who preferred prevention of hospital associated infections blamed the NHS, a majority focused on the issue of trust. Respondents primarily indicated that they felt it was the NHS’ responsibility to provide safe and effective care facilities. Some prioritised hospital associated infection because they found the NHS not fulfilling its duty to be unacceptable and a few stated that this would damage the credibility of the institution:

(ID 1886) hospital superbugs are a hot topic in the public perception of a hospital safe and trusted environment so any case is a news worthy event if a patient suffers due to poor hygeine in hospitals, so, the perceived impact is greater than the statistics quoted as the hospital patient/ carer trust is damaged.

(ID 426) it is totally wrong to be worse off by being in a care facility.

They further noted that if someone enters a hospital, it is because they need medical attention, and that catching a new infection while in the hospital would only aggravate their health problems. Some respondents mentioned additional costs in the long run due to potential litigation. Dread or fear of hospital associated infection was also mentioned a few times and was unique to this context.

About twelve percent of the responses challenged the assumption of equal health loss avoided. Some of them commented that hospital associated infections have greater likelihood and others stated that they are more severe.

### Preferred preventing medication errors

Almost 30% of the respondents mentioned NHS system or staff member’s fault as the reason for preferring medication errors, for example:

(ID 3131) I believe negligence and avoidable harm is most tragic, thus that should be prevented foremost in my opinion.

(ID 1144) there is no excuse for trained staff to give patients the wrong drugs.

The issue of trust in the NHS was the second most cited reason in this category, with respondents saying that the healthcare system has a duty to provide safe and effective treatment to patients. It was also mentioned that it is unacceptable for the NHS to be causing harm and that patients should be able to trust the system. A few responses also mentioned litigation charges attached to NHS caused adverse events.

The assumption of equal health gain from both contexts was again challenged. A total of 9% of respondents differentiated on the basis of severity of the condition. Most of them mentioned that medication errors are dangerous and can be fatal. Few responses cited that genetic disorders were more prevalent than medication errors.

### Prefer preventing staff injury

A majority of comments in this group indicated that it is the NHS’s duty as an employer to look after its employees –

(ID 3402) NHS is reponsale for the well being of there staff.

(ID 6481) everyone has the right to be safe in their work place environment.

Concern for future considerations was also cited, where excerpts indicated a reduction in the NHS’s ability to care for patients if NHS staffs are injured, for example:

(ID 2972) Unless invaluable NHS staff are given maximum protection there won’t be an NHS.

(ID 1834) we have to ensure nhs staff are protected otherwise there would be no staff left or wanting to work to look after us.

(ID 1062) i Chose A because the workers need to be valued. without them patients cannot be looked after which is not good for the system.

Some of the respondents prioritising prevention of genetic disorder over staff injuries disagreed with the assumptions stated in the survey. For example some comments indicate that they thought staff injuries were less prevalent or less serious than genetic disorder.

### Comparisons with other contexts

Examination of comparison of contexts with similar responsibility shed further light on the perception of responsibility and the acceptance of hypothetical choice sets. In the direct comparison of lifestyle related diseases to sports injuries, many respondents noted that both were self-inflicted as expected. Some viewed sports as a choice while others viewed that sports injuries as accidents. However a relatively large number of respondents considered sports injuries less severe and less prevalent than lifestyle diseases, and did not prioritise them. Nevertheless there were a few who prioritised prevention of sports injuries over lifestyle related because “*people do sports to remain healthy so more should be done for these people than people who smoke etc.*”.

In the comparisons between healthcare associated infections and medication errors, a majority of respondents viewed both as equal and important, with most mentioning that both are NHS’s responsibility. When the same contexts were compared against staff injuries, the respondents made a distinction between services for patients and staff, and were biased towards patients.

ID 1815 A patient can have no control over which drugs are administered and if given wrong drugs then this would be an NHS problem. Injuries in the NHS workplace are easily control by higher health and safety standards, less overcrowding, shorter working hours etc.

ID 1867 because people choose to work in a hospital and take that risk, people do not choose to catch an infection.

Also hospital associated infections were considered more prevalent, affecting more people in one incident than medication error or staff injuries. For example:

(ID 1643) infection spreads rapidly whereas injuries can be just a few individuals.

(ID 966) The first option is for all who attend hospital and so would affect a wider range of people.

## Discussion

Our study analysed free text comments provided by respondents explaining their preferences over interventions to prevent harm from lapses in healthcare safety and patient lifestyle choices. A majority of the 1030 respondents chose to comment although they had the option to not comment. We had a large qualitative dataset; however the comments provided were brief as they were collected as part of a valuation survey and in-depth analysis was not possible. Nevertheless we were able to shed light on factors considered when comparing lapses in healthcare safety and patient lifestyle choices, as well as to validate our results.

Most stated preference methods present hypothetical scenarios to elicit preferences from the general public. In order to reduce cognitive burden, many assumptions need to be made. The choice questions in our study asked for a choice between preventative services which were framed as identical in terms of costs and effects, in order to encourage respondents to trade off one context against another purely in terms of the number of persons affected. We repeatedly stated that everything else was held constant, however examining the comments revealed that some respondents challenged these assumptions. These excerpts explicitly rejected the assumptions or misinterpreted the cost and consequences associated with the services. As for other respondents who did not explicitly challenge these assumptions in the comments provided, it is still not possible to confirm if they accepted the equal cost and benefit assumption while making their choices. Nevertheless, re-analysing the quantitative results after excluding responses and respondents who challenged the PTO assumptions made little difference. Perhaps allowing for face to face administration of PTO questions would have helped in finding out whether the challenges to the assumptions were due to not absorbing the information provided or because they simply disagreed with the assumptions.

There were a few reasons raised by the respondents which could be interpreted as a rejection of the assumptions underlying the choice questions, for example competing context should be addressed using separate funding (non-NHS), inevitability (with reference to the illness) or preventability (with reference to the nature of intervention, for example a service considered treatment related rather than prevention). These comments were categorised as codes explaining preferences within the framework rather than as challenges to the question assumptions. The codes were attributed in accordance with the process of the two coders operating separately. We do however recognise that some of these could be interpreted as challenges to the assumptions. This reflects the subjective nature of the interpretation of the comments, which is inevitable in this type of analysis.

The survey found that members of the general public attach a premium to health loss due to some types of lapses in healthcare safety but not to all. Hospital associated infections were given a premium but medication errors were rated similar to genetic disorders, and staff injuries were given less weight than genetic disorders. NHS culpability and NHS duty to provide safe, clean and effective care were the most cited reasons for preferring healthcare safety contexts. However there were also respondents who did not prioritise such lapses because they could have been avoided with better training or better staff. Some also said that it was fundamental that the NHS should fulfil its duty of care, and that services to prevent lapses in NHS safety should already be in place or be paid for elsewhere in the system. If these reverse preferences were to be taken into account, the premium attached to hospital associated infections and medication errors could be even greater. Many also differentiated these contexts from the basecase saying that those who are going to be affected by healthcare adverse events are already vulnerable or in a poor health state. In the case of staff injures, it is important to note that only this context related to staff rather than patients safety. Although similar reasons were provided for preferences for NHS healthcare safety contexts, hospital associated infections were mostly associated with an NHS duty of care (trust), medication errors were attributed to NHS staff/system culpability, while NHS staff safety was associated primarily with workplace responsibility.

Health loss due to a person’s own action was generally given lower priority. Preventing lifestyle disease and sports injuries were said to be either partially or wholly the responsibility of the individual. However participants frequently mentioned that sports injuries are less severe and less likely to occur than genetic disorders.

A majority of responses put forward an extra consequentialist case, where considerations other than direct costs and health consequences were cited to explain preference between contexts. Overall responsibility was cited as the main differentiating factor and comprised 75% of the total responses which is not surprising as the study was designed to differentiate contexts based on responsibility. Other considerations which were cited related to the nature of the disease or injury, patient characteristics and wider impact. In terms of nature of the health loss - inevitability of the illness or injuries, and preventability were indicated in many of the excerpts. For example, genetic disorders were considered unavoidable suggesting that respondents associated them with treatment services rather than preventive services. As future research, it would be interesting to explore whether the public still differentiate between services on the basis of responsibility when the services are presented as treatment rather than preventative interventions.

The findings of this study are comparable to those of the study by Steuten and Buxton (2010), in which healthcare professionals were interviewed to identify the most important attributes of healthcare safety [14]. They concluded that preventability of healthcare incidents, health consequences, financial consequences and trust in safety systems/devices were the most valued attributes of healthcare safety. Although healthcare costs and consequences were held constant in our study, preventability and trust were mentioned. There were four additional attributes that were listed in the Steuten and Buxton paper, which emerged in our analysis: likelihood of the event, voluntariness of being in a risky situation (individual responsibility), dreadfulness and equality.

## Conclusion

By collecting open ended comments in an online stated preference survey, we have been able to provide an ‘explanatory adjunct’ to understand public preferences regarding lapses in healthcare safety and lifestyle diseases [15]. The comments show that many people do have preferences over the prioritisation of NHS resources related to the perceived ‘responsibility’ for the health condition, but that these preferences are heterogeneous and complex. The comments were also used to verify if the assumptions presented in the hypothetical choice questions were accepted by the respondents. We found that, although many respondents made comments that suggested that they did not always accept the assumptions, excluding these responses or respondents did not change the overall results.

Most stated preference methods use hypothetical scenarios to elicit preferences and it is important to verify that the respondents understand and process the question in the way that is intended. Analysis of free text comments can help with this verification process, as well as shedding light on the biases and other factors that the respondent may have added to the information provided while making their choice. The focus of stated preference studies should be on not just quantifying public preferences but also getting a better understanding of the underlying reasons for people’s stated preferences.

## Competing interests

The authors declare that they have no competing interests.

## Authors’ contributions

MJB conceived the study along with JL, LL, JS and SO. AB and JS conducted qualitative analysis of the data with guidance from LL. JS drafted the manuscript with help from LL and JL. All authors contributed to critical revision of the manuscript and made a substantial contribution to its contents. All authors have read and approved the final manuscript.

## Pre-publication history

The pre-publication history for this paper can be accessed here:

http://www.biomedcentral.com/1472-6963/13/249/prepub
